# Rally and recover: Physiological demands between tennis drills

**DOI:** 10.1371/journal.pone.0340767

**Published:** 2026-01-12

**Authors:** Glenn Björklund, Mikael Swarén, Fredrik Johansson

**Affiliations:** 1 Swedish Winter Sports Research Centre, Department of Health Sciences, Mid Sweden University, Östersund, Sweden; 2 Swedish Unit for Metrology in Sports, Department of Sports and Health Sciences, School of Health and Welfare, Dalarna University, Falun, Sweden; 3 Sophiahemmet University, Stockholm, Sweden; 4 Scandinavian College of Manual Medicine, Stockholm, Sweden; İzmir Democracy University: Izmir Demokrasi Universitesi, TÜRKIYE

## Abstract

This study examined physiological recovery between repeated tennis drills in elite adolescent tennis players. Ten tennis players (5 males, 5 females; age 17 ± 2 years) underwent treadmill testing to establish maximal physiological characteristics. Several days later participants completed three standardized on-court tennis drills with fixed rest intervals, during which physiological parameters were monitored. A one-way repeated-measures ANOVA was used to compare physiological responses across the three drills. $\dot{\text{V}}{\text{O}}_{\text{2max}}$ utilization stayed above 75% in all drills, peaking during the first drill (p = 0.003). During recovery, $\dot{\text{V}}{\text{O}}_{\text{2max}}$ utilization decreased from 58 ± 8% in the first recovery to 50 ± 9% (p = 0.018) and 47 ± 12% (p = 0.022) in the second and third recovery, respectively. The respiratory exchange ratio (RER) stayed below 1.0 during drills, while increasing during recovery periods, (1.07 ± 0.08, 1.00 ± 0.01, 1.04 ± 0.05; p = 0.014). Ventilatory equivalents for oxygen ($\dot{\text{V}}\text{E}/{\dot{\text{V}}\text{O}}_{\text{2}}$) were stable (p = 0.054), while those for carbon dioxide ($\dot{\text{V}}\text{E}/{\dot{\text{V}}\text{CO}}_{\text{2}}$) increased progressively for each recovery period (29.5 ± 3.6, 31.5 ± 3.8, 32.3 ± 4.3; p < 0.001). Blood lactate concentration differed significantly across recovery periods (p = 0.035) with the lowest value in recovery period two (5.9 ± 2.0, 4.9 ± 1.9 and 5.6 ± 2.0 mmol·l^-1^). These findings highlight a sustained metabolic demand extending into the recovery phase during standardized tennis drills, characterized by substantial $\dot{\text{V}}{\text{O}}_{\text{2max}}$ utilization and elevated respiratory compensation. This suggests a significant anaerobic contribution and underscores the intensity of the physiological load imposed even after exercise cessation.

## Introduction

Tennis involves intermittent activity relying on both aerobic and anaerobic energy systems [1–3]. Match intensity is shaped by factors such as rally duration, playing surface, stroke type, and stroke frequency [4–7]. Additionally, the duration of rest between rallies significantly affects both physiological strain and stroke quality [8]. Tennis-specific field testing has demonstrated that rally density, expressed as stroke or ball frequency, is closely linked to metabolic and ventilatory stress. Strong associations have been reported between ball frequency, oxygen uptake, and the second ventilatory threshold during progressive on-court tennis exercise [9]. Although average oxygen uptake during tennis matches is generally low to moderate, periods of intense rallying can exceed external demands that surpass maximum oxygen uptake ($\dot{\text{V}}{\text{O}}_{\text{2max}}$), involving a substantial anaerobic contribution [10]. Given these physical demands incorporating match-replication drills into training is essential for effective conditioning. Previous studies have examined the physiological demands and recovery associated with tennis drills using internal load markers such as heart rate, blood lactate concentration, and ratings of perceived exertion (RPE) [8,11]. Despite advances in monitoring physiological responses during tennis practice, data on oxygen uptake during recovery phases between repeated drills are still scarce. In swimming, for example, post-exercise oxygen uptake (EPOC) in combination with blood lactate measurements, has been employed to quantify the anaerobic contribution over various race distances [12]. Therefore, fundamental data on oxygen uptake during recovery between repeated tennis drills could add valuable insights of the physiological strain. In intermittent sports as tennis supramaximal efforts elevate EPOC due to the need to restore oxygen stores, resynthesize ATP and phosphocreatine, normalize pH, and support ventilatory demands [13,14]. Another approach to assessing exercise intensity and physiological strain is the ventilatory responses, particularly the ventilatory equivalents $\dot{\text{V}}\text{E}/{\dot{\text{V}}\text{O}}_{\text{2}}$ and $\dot{\text{V}}\text{E}/{\dot{\text{V}}\text{CO}}_{\text{2}}$ [15–17]. An increase in $\dot{\text{V}}\text{E}/{\dot{\text{V}}\text{O}}_{\text{2}}$ typically signals the shift from low- to medium-intensity exercise, while the additional increase in $\dot{\text{V}}\text{E}/{\dot{\text{V}}\text{CO}}_{\text{2}}$ marks the transition from medium- to high-intensity exercise. While ventilatory responses are well-studied during incremental and constant work rate protocols, there are sparse data from variable intensity exericse [18,19]. Previous research has indicated that tennis drills replicate the physiological demands of normal to maximal match play, albeit with some variation in stress levels between drills [11,20]. Therefore, tennis drills can be considered a valid method for replicating actual match play. Although previous research has effectively described the average oxygen uptake during tennis practice, the resting periods between drills and series of tennis strokes have not been investigated separately. Overall, data from previous studies suggest that tennis drills incorporate high-intense elements based on heart rate and blood lactate concentration. However, no study to date has investigated the magnitude or potential build up of physiological strain between repeated tennis drills regards oxygen uptake or respiratory compensation. Consequently, investigation of the respiratory responses during rest periods in tennis could further provide insights of the acute physiological recovery during tennis practice.

The aim of this study was to assess and characterize the rest periods following repeated tennis drills, with a particular focus on of oxygen uptake ($\dot{\text{V}}{\text{O}}_{\text{2}}$), ventilatory responses ($\dot{\text{V}}\text{E}/{\dot{\text{V}}\text{O}}_{\text{2}}$ and $\dot{\text{V}}\text{E}/{\dot{\text{V}}\text{CO}}_{\text{2}}$) and their recovery dynamics.

## Materials and methods

### Participants

Ten adolescent elite tennis players from the Swedish national teams participated in the study (Table 1). The recruitment period for this study commenced on 25/11/2018 and concluded on 19/01/2019. The players competed at the International Tennis Federation (ITF) level and classified as tier 3 athletes according to participant classification framework [21]. Exclusion criteria included recent injuries and/or illnesses requiring more than a week’s rest from training or competition in the past three months, or sickness for more than three days in the last four weeks prior to the aerobic and anaerobic tests. All players were right-handed and used a two-handed backhand. Their weekly training volume varied by age, ranging from 12 to 20 hours, in accordance with national recommendations set by the Swedish Tennis Association for elite adolescent players. This time includes match play, with a total of 100–120 matches played annually at both national and international level. Prior to participation, participants received full explanations of procedures and potential risks. Written informed consent was obtained from all participants and their legal guardian or next of kin before the experiments began. The study design was pre-approved by the Regional Ethical Review Board, Stockholm, Sweden (approval no. 2012/1731/2).

**Table 1:** Demographic data and physiological characteristics.

	Mean ± SD
Males/females (no.)	5/5
Height (cm)	179 ± 9
Body mass (kg)	71 ± 9
$\dot{\text{V}}{\text{O}}_{\text{2max}}$ (l·min^-1^)	3.8 ± 0.6
$\dot{\text{V}}{\text{O}}_{\text{2max}}$ (ml·kg^-1^·min^-1^)	54 ± 6
BLa_max_ (mmol·l^-1^)	12.1 ± 1.2
HR_max_	199 ± 5
Inclination_max_ (%)	7.7 ± 0.7
Speed_max_ (km·h^-1^)	13 ± 1
RPE Overall (6–20)	20 (19, 20)*
RPE Legs (6–20)	20 (19, 20)*

$\dot{\text{V}}{\text{O}}_{\text{2max}}$, maximum oxygen uptake; BLa_max_, maximum blood lactate concentration; Inclination_max_, maximum inclination; Speed_max_, maximum speed; RPE, rating of perceived exertion. All physiological variables were measured during an incremental maximal treadmill running test. *RPE (6–20) is presented as median (IQR).

### Laboratory tests

A maximal laboratory treadmill test was conducted for each player to establish the players physiological characteristics (Table 1). Before the test was initiated a progressive 10-min warm-up on a treadmill (Katana, Lode, Groningen, the Netherlands) was performed followed by a 3-min rest period. The running speed was individually adjusted for each player during the warmup according to previous self-reported test results. A predetermined constant running speed was used throughout the test while the workload increased by inclination in 1-minute steps, 2% at the 1-minute mark, and subsequently by 1% until the athlete experienced voluntary exhaustion. Blood lactate concentration was determined one- and three-minutes post-test through capillary blood samples. Capillary blood samples were collected from the nondominant hand to determine blood lactate concentration with the first drop of blood discarded. A 20 µl sample was drawn using a capillary tube and analyzed with a Biosen C-Line analyzer (EKF Diagnostics, Magdeburg, Germany). Expired gas and ventilation were continuously measured using a metabolic cart (Jaeger Oxycon Pro, Wuerzburg, Germany) in mixing chamber mode. Before each test, the gas analysers turbine was calibrated. $\dot{\text{V}}{\text{O}}_{\text{2max}}$ was considered achieved if two of the following three criteria were met: (1) $\dot{\text{V}}{\text{O}}_{\text{2}}$ plateau with an increase less than <150 ml·min^-1^, (2) respiratory exchange ratio exceeded 1.10, and (3) maximal blood lactate samples > 8 mmol·l^-1^. The highest $\dot{\text{V}}{\text{O}}_{\text{2}}$ averaged over a period of 60 s was used to calculate $\dot{\text{V}}{\text{O}}_{\text{2max}}$. Immediately post-exercise, the Borg Rating of Perceived Exertion (RPE) scale (range: 6–20) was administered to quantify players subjective perception of overall and leg physical exertion.

### Field tests

#### Tennis drills.

Before the field test, players completed a standardised 15 min warm up on a cycle ergometer (LT2, Monark Exercise AB, Vansbro, Sweden) set at 2 W per kilo body mass for the males and 1.5 W per kilo body weight for the women. After this cycling warm up, players engaged in five minutes of individual dynamic movements and stretching, followed by ten minutes of hitting warm up. Participants completed three randomized tennis drills (Drill 1–3), each consisting of eight sets of 3–6 strokes lasting 10–15 seconds per set described by Björklund et al [22]. In addition, the drills were modelled on previously validated on-court tennis drill protocols with specified durations, repetitions, and pacing designed to reflect match-play dynamics [11,23]. A detailed overview of drill structure, duration, repetitions, external pacing cues, and external load parameters is provided in Table 2. In the present study, recovery was assessed following the first three consecutive drills using a portable breath-by-breath gas analyzer and blood lactate sampling. The rest period following each drill was set to 90 seconds to replicate the maximum duration between consecutive games, representing the changeover phase. An experienced professional coach, standing in the center of the court following the player, hand-fed new tennis balls to the player at a speed determined by the completion of the previous shot and movement of the player to the next shot, i.e., self-selected [11,23]. Ball placement followed a fixed order presented to the players in advance at a frequency of approximately one ball every three seconds. Each drill was based on movement patters seen in match play and designed by one fitness coach and one ATP coach in agreement. All players were instructed to move as quickly as possible during the drills and to hit the ball with maximum effort, ensuring the ball remained in play. After completing each drill the players returned walking to the centre mark of the court behind the baseline. All testing was conducted indoors under stable environmental conditions, with temperature and humidity remaining consistent across testing sessions.

**Table 2:** External load overview.

Metric	Value
Ball Feed Rate	~1 ball every 3 s
Strokes per set	3 - 6
Set Duration	10–18 s
Average speed	2.2 m·s^-1^
Rest between sets	25 s
Total Sets per Player	24 (8 per drill × 3 drills)
Total Strokes per Player	104 (48 + 32 + 24)
Total average distance Per Player	77.3 m
Effort Level	Maximal
Drill Design Basis	Match-play movement patterns

m, meters; meters per second, m·s^-1^; s, seconds. Average values represent the mean of the data.

### Oxygen uptake and blood lactate

All tennis players were fitted with a portable breath-by-breath gas analyser (MetaMax3B_R2; Cortex Biophysik GmbH, Leipzig, Germany). The sampling frequency analyzed were breath-by-breath and not through any set interval in seconds. The gas analysers were calibrated between the tests using a two-point method for oxygen (${\text{O}}_{\text{2}}$) and carbon dioxide ($\text{C}{\text{O}}_{\text{2}}$) sensors, with ambient conditions and a mixture of 15% ${\text{O}}_{\text{2}}\;$and 5% $\text{C}{\text{O}}_{\text{2}}$ (UN 1950 Aerosols, Cortex Biophysik GmbH, Leipzig, Germany). The turbine flow was pre checked with a 3 L syringe (M9474-C, Medikro Oy, Kuopio, Finland). The analyser was securely attached to minimise disruption to normal play, and the face mask was positioned to avoid obstructing players’ vision. Capillary blood samples were obtained from the nondominant hand to determine blood lactate concentration. Baseline blood lactate concentration was measured prior to Drill 1, with additional samples collected immediately after each completed drill. Before sampling, the fingertip was disinfected using an antiseptic solution (Klorhexidin, Fresenius Kabi AB). A 20 µl blood sample was drawn using a capillary tube and transferred into a pre-filled Safe-Lock reaction cup, with the first drop of blood discarded. All post-exercise samples were collected within 30 s of drill completion and subsequently analyzed using a Biosen C-line analyzer (EKF Diagnostics, Magdeburg, Germany).

### Statistical analysis

Data are presented as mean and standard deviation (SD). Data were analysed using JASP [24]. All data were pre-checked for normal distribution with Shapiro Wilk test before further analysis. Cardiorespiratory data collected during the recovery periods between stroke series were analyzed using a one-way repeated-measures ANOVA. Early-to-late recovery analysis of $\dot{\text{V}}{\text{O}}_{\text{2}}$ was conducted by comparing the mean values of the first four breaths and the last four breaths within each recovery period. Effect sizes were quantified using omega squared (ω²), with benchmarks of 0.01, 0.06, and ≥0.14 representing small, medium, and large effects, respectively [25]. For all ANOVA tests, Mauchly’s sphericity test was conducted to control for Type I errors. If the assumption of sphericity was violated, the Greenhouse-Geisser correction was applied to adjust the F values. If there were global significances for the ANOVA a Bonferroni Post Hoc analysis was conducted with Cohen’s *d* for pairwise effect size. Thresholds of 0.2, 0.5, and ≥0.8 denoting small, medium, and large effects, respectively [26]. A Pearson correlation coefficient (r and 95% confidence interval [lower limit, upper limit]) was performed to evaluate relation between$\;\dot{\text{V}}{\text{O}}_{\text{2max}}$ and blood lactate concentration. The α was set a priori to < 0.05.

## Results

### Tennis drills and Work:Rest (W:R) ratio

The time durations for the tennis drills were for drill 1–3; 307 ± 28 s, 261 ± 23 s, and 294 ± 64 s (F_2,18_ = 4.78, p = 0.022, ⍵^2^ = 0.140). Drill 2 was the shortest of the three and significantly different to bout 1 (p = 0.006, Cohen’s *d* = 1.080). The duration of the rest periods after tennis drill 1–3 were 94 ± 18 s, 94 ± 20 s, and 89 ± 28 s, respectively. There was no difference in duration for rest periods (F_2,18_ = 0.21, p = 0.815, ⍵^2^ < 0.001). Furthermore, the W:R ratio was not significantly different between 1–3: 3.37 ± 0.78, 2.91 ± 0.75 and 3.71 ± 1.86 (F_2,18_ = 1.09, p = 0.355, ⍵^2^ = 0.006).

### $\dot{𝐕}{𝐎}_{2}$ and percent utilization during drills

The average $\dot{\text{V}}{\text{O}}_{\text{2}}$ during the exercise bouts differed (F_2,18_ = 7.97, p = 0.003, ⍵^2^ = 0.024) and were greatest during the first exercise bout (3.11 ± 0.48 l/min) in comparison to the second (2.94 ± 0.45 l/min, p = 0.008, Cohen’s *d* = 0.363) and third exercise bout (2.94 ± 0.53 l/min, p = 0.009, Cohen’s *d* = 0.355). There was no difference in $\dot{\text{V}}{\text{O}}_{\text{2}}$ between bout 2 and 3 (p = 1.000, Cohen’s *d* = 0.008). Percent usage of $\dot{\text{V}}{\text{O}}_{\text{2max}}$ during the different drills were 81.5 ± 5.5%, 76.9 ± 5.3%, 77.0 ± 7.1% (F_2,18_ = 6.81, p = 0.006, ⍵^2^ = 0.097). Percent usage of $\dot{\text{V}}{\text{O}}_{\text{2max}}$ was greatest during drill 1 (p = 0.013, Cohen’s *d* = 0.763 and p = 0.013, Cohen’s *d* = 0.746, vs drill 2 and 3 respectively). RER remained at the same level for all exercise bouts (0.95 ± 0.04, 0.95 ± 0.02 and 0.95 ± 0.02, for 1–3 respectively, F_2,18_ = 0.28, p = 0.755, ⍵^2^ = 0.001).

### $\dot{𝐕}{𝐎}_{2}$ and percent utilization during recovery

**Fig 1:**
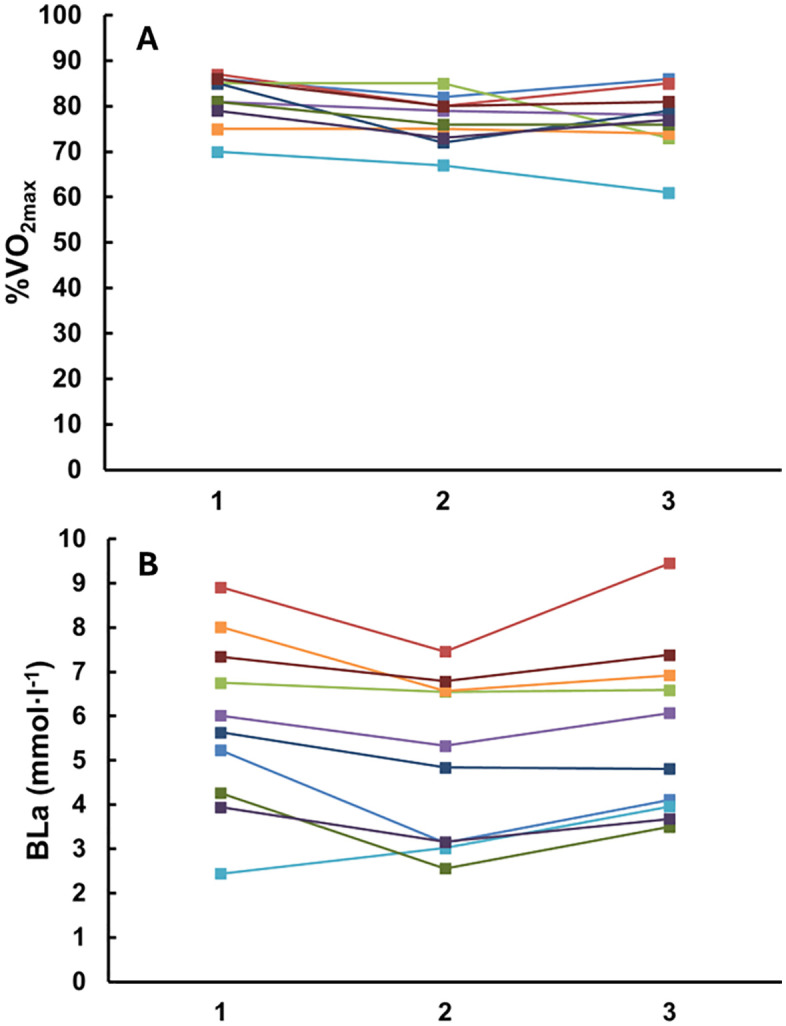
Individual trajectories for: A) Percent utilization of maximal oxygen uptake ($\begin{array}{c} \dot{V}O_{2max} \end{array}$) throughout the recovery period following drills 1–3 and B) Blood lactate concentration (BLa; mmol·l  ⁻ ¹) measured post drills 1–3. The duration of the recovery periods 1–3 were on average 94, 94 and 89 s respectively.

**Fig 2:**
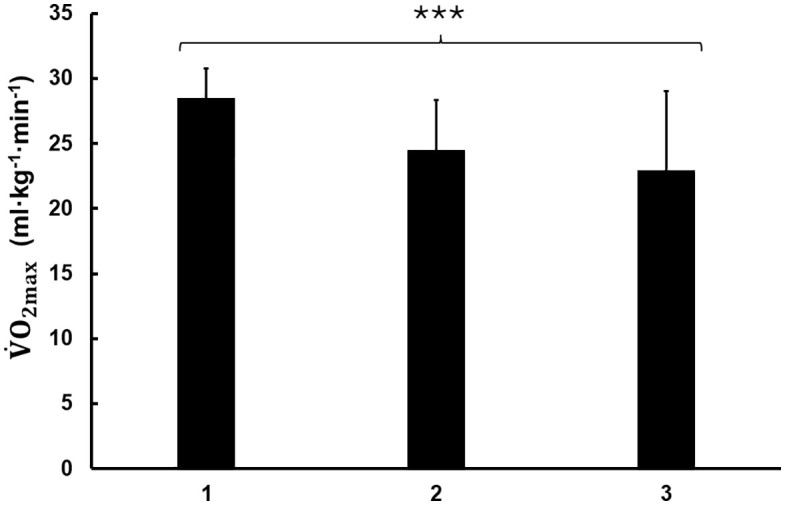
Oxygen uptake during recovery. Relative maximal oxygen uptake ($\dot{\text{V}}{\text{O}}_{\text{2max}}$) expressed as ml·kg^-1^·min^-1^ for recovery periods post drills 1–3 presented as mean ± SD. A statistically overall significant difference between recovery periods 1 to 3 is indicated by *** p < 0.001. The duration of the recovery periods 1–3 were on average 94, 94 and 89 s respectively.

The percent use of $\dot{\text{V}}{\text{O}}_{\text{2max}}$ decreased during the consecutive recovery periods (F_2,18_ = 7.43, p = 0.004, ⍵^2^ = 0.165, Fig 1A). During the first recovery period the utilisation of $\dot{\text{V}}{\text{O}}_{\text{2max}}$ was the greatest (58 ± 8%) compared to both the second (50 ± 9%, p = 0.018, Cohen’s *d* = 0.800) and third recovery period (47 ± 12%, p = 0.022, Cohen’s *d* = 1.139). There were no significant differences between recovery period two and three (p = 1.000, Cohen’s *d* = 0.339). Relative $\dot{\text{V}}{\text{O}}_{\text{2}}$ expressed as ml·kg^-1^·min^-1^ was greatest during recovery period 1 in comparison to recovery 2 and 3 (F_2,18_ = 6.99, p = 0.008, ⍵^2^ = 0.197, Fig 2). The reduction in relative $\dot{\text{V}}{\text{O}}_{\text{2}}$ (ml·kg^-1^·min^-1^) from early to late recovery was observed across the three recovery periods as follows: 17 ± 9, 13 ± 10, and 8 ± 8 for periods 1 through 3, respectively. Statistical analysis revealed no significant differences between periods (F_2,18 _= 2.34, p = 0.125, ω² = 0.074).

When expressed as a percentage change, the early to late recovery decrease in $\dot{\text{V}}{\text{O}}_{\text{2}}$ was −41 ± 21%, −34 ± 25%, and −26 ± 21% for recovery periods 1–3, respectively. This difference was also not statistically significant (F_2,18 _= 1.31, p = 0.294, ω² = 0.017).

### Respiratory variables

Overall, RER values remained over 1.0 for all three recovery periods but was greatest during recovery 1 (Table 3). Compared to recovery 1, the lowest RER values were during recovery 2 (p = 0.028, Cohen’s *d* = 1.053).

**Table 3:** Respiratory variables and blood lactate concentration during recovery periods.

	Recovery 1	Recovery 2	Recovery 3	ANOVA (F-, p-value, ⍵^2^)
RER	1.07 ± 0.08	1.00 ± 0.01*	1.04 ± 0.05	F_2,18_ = 5.51, p = 0.014, ⍵^2^ = 0.132
$\dot{\text{V}}\text{E}/{\dot{\text{V}}\text{O}}_{\text{2}}$	31.6 ± 4.7	31.6 ± 3.9	33.7 ± 5.4	F_2,18_ = 3.74, p **= **0.054, ⍵^2^ = 0.030
$\dot{\text{V}}\text{E}/{\dot{\text{V}}\text{CO}}_{\text{2}}$	29.5 ± 3.6†	31.5 ± 3.8*	32.3 ± 4.3*	F_2,18_ = 20.50, p < 0.001, ⍵^2^ = 0.078
BLa (mmol·l-1)	5.9 ± 2.0	4.9 ± 1.9*†	5.6 ± 2.0	F_2,18_ = 8.49, p = 0.003, ⍵^2^ = 0.035

Data is presented as Mean ± SD during the recovery periods.

RER, respiratory exchange ratio; $\dot{\text{V}}\text{E}/{\dot{\text{V}}\text{O}}_{\text{2}}$, ventilatory equivalent of oxygen uptake; $\dot{\text{V}}\text{E}/{\dot{\text{V}}\text{CO}}_{\text{2}}$, ventilatory equivalent of carbon dioxide production; BLa, blood lactate concentration.

*Significantly different from Recovery 1 (p < 0.05).

†Significantly different from Recovery 3 (p < 0.05).

The $\dot{\text{V}}\text{E}/{\dot{\text{V}}\text{O}}_{\text{2}}$ ratio remained on a high level for all recovery periods with no significant change (Table 3). In contrast, $\dot{\text{V}}\text{E}/{\dot{\text{V}}\text{O}}_{\text{2}}$ was lowest at the first two recovery periods but increased for recovery period 3 (p = 0.034, Cohen’s *d* = 0.440, Table 3). There was no difference between recovery 1 and 2 (p = 1.000, Cohen’s *d* = 0.010).

### Blood lactate concentration

Pre-exercise blood lactate concentration was 1.5 ± 0.4 mmol·l^-1^. Blood lactate concentrations remained elevated throughout recovery periods, with a significant drop during the second recovery period compared to the first (p = 0.015, Cohen’s *d* = 0.471, Table 3). A significant increase in blood lactate concentration was observed during the third recovery period (p = 0.011, Cohen’s *d* = 0.365). Fig 1B highlights the considerable inter-individual variability in lactate responses across the different recovery periods. There was no relationship to blood lactate concentration at the rest periods and $\dot{\text{V}}{\text{O}}_{\text{2max}}$, (ml·kg^-1^·min^-1^), (*r* = −0.159 [0.-717, 0.523], p = 0.661) (*r* = −0.075 [−0.673, 0.582], p = 0.838) (*r* = −0.070 [−0.670, 0.585], p = 0.847).

## Discussion

To our knowledge this is the first study that has studied the cardiorespiratory effects during recovery periods between high intense tennis drills. The major finding is the substantial average oxygen uptake during the 90 s recovery phase that’s approximately 50 percent of the players $\dot{\text{V}}{\text{O}}_{\text{2max}}$. The second finding is the sharp increase in respiratory exchange ratio (RER) that increases > 1.0 during the recovery phase although it remains < 1.0 during all rallies. In addition, ventilatory equivalent for oxygen uptake is already at a high level after the first bout which shows a shift towards anaerobic energy contribution from the start. Furthermore, the increase in ventilatory equivalent of carbon dioxide for the second and third recovery period marks that the players has crossed the respiratory compensation point, i.e., a transition from the buffering to a hypercapnic state induced by metabolic acidosis.

### Cardiorespiratory response

The fact that $\dot{\text{V}}{\text{O}}_{\text{2}}$ remained above 50% between drills clearly proves that the resting periods involves recovery processes demanding an increased need for oxygen involving restoration of high-energy phosphagens, lactate removal and heat dissipation. Furthermore, such high levels of oxygen uptake between exercise bouts do not resemble a recovery pattern from medium intensity exercise rather high intensity exercise. Importantly, the study design is aimed to mimic rest between games and not rallies. However, most certainly the games vary much more than mimicked in current study [27]. Nevertheless, one important aspect to study the recovery periods were to illustrate the how intense tennis drills are. Although it’s certainly possible to add aerobic training afterward, it is more likely to be low-intensity exercise due to the anaerobic demands involved in performing tennis drills such as the ones described in the current study. In line with the blood lactate concentration variation, RER followed the same pattern in mean values. The sustained elevation in respiratory compensation observed across all recovery periods may reflect a continued physiological effort to buffer metabolic acidosis, potentially linked to the limited reduction in blood lactate concentrations [28]. The coupling with respiratory compensation and metabolic acidosis has also been confirmed during variable intensity [29]. During submaximal exercise intensities, RER serves as an indicator of substrate utilization, primarily reflecting the relative contributions of carbohydrates and lipids to energy metabolism. However, post supramaximal exercise RER primarily reflects respiratory compensation to maintain a neutral pH. Additionally, $\dot{\text{V}}\text{E}/{\dot{\text{V}}\text{O}}_{\text{2}}$ and $\dot{\text{V}}\text{E}/{\dot{\text{V}}\text{CO}}_{\text{2}}$ are often used to determine intensity zones as low medium and high [30]. Values exceeding 30 are generally considered indicative of high intensity physiologiocal effort. Although the present study did not specifically investigate the ventilatory equivalents during exercise, but rather during recovery, the ventilatory equivalent of $\dot{\text{V}}\text{E}/{\dot{\text{V}}\text{CO}}_{\text{2}}$ suggest that a pronounced ventilatory drive may have persisted throughout recovery, possibly as a response to ongoing metabolic acidosis. Interestingly, previous work in roller-ski skating shows that ventilatory thresholds may be obscured when breathing is mechanically constrained by upper-body-dominant movements [31]. Similarly, in tennis, stroke mechanics and trunk stabilization may constrain ventilation, warrant caution when interpret ventilatory markers alone. In this context, the heart rate deflection point and corresponding ball tempo from on-court tests provide practical alternatives that closely approximate VT2, ($\dot{\text{V}}\text{E}/{\dot{\text{V}}\text{CO}}_{\text{2}}$and allow individualized training prescription using ball tempo [9]. It is possible that the metabolic cart was unable to accurately detect the abrupt shift toward anaerobic metabolism occurring during tennis practice. However, this explanation is tentative and should be interpreted with due caution. In summary, these findings underscore the importance of respiratory markers an elevated oxygen uptake in understanding recovery dynamics during tennis practice.

### Blood lactate recovery

The blood lactate concentration response in our study confirms previous study results using similar set-up for characterisation of the physiological demands of tennis practice [2,5,11]. Interestingly, while the blood lactate concentration remains clearly above resting values the second recovery period has the lowest blood lactate concentration of the three recovery periods. Accordingly, the lowest blood lactate concentration during the second recovery period shows less anaerobic contribution between the drills. This is aligned with the respiratory exchange ratio which also shows it lowest value during the second recovery period. However, the coupling with elevated blood lactate concentration and post oxygen consumption in current study do not confirm studies using only one bout with various exercise intensities. Likely there is a carryover effect from previous tennis drill using repeated bouts as the third recovery period shows similar blood lactate concentration as the first recovery period while the oxygen uptake is clearly reduced. Consequently, if the player has increased blood lactate concentration after the first bout it remains high for the consecutive exercise bouts. This highlights the difficulties to get rid of a previous accumulated high blood lactate concentration between rallies. If the plan is to remain low in blood lactate concentration between tennis drills, active recovery is superior to passive recovery [32]. However, the time span for blood lactate concentration to show a reduction even with active recovery is far longer than the ~ 90 s recovery period used in current study. While a possible scenario would be to change from passive to active recovery in tennis for blood lactate recovery, the time frame is too short as it seems. Moreover, the suggestion to include active recovery at a work rate that coincide with the individual lactate threshold is problematic in tennis [33]. The difficulties to use the external work rate is very complicated in tennis although possible, so it’s not realistic to use in tennis practice between drills. On the other hand, if there is a longer break between sessions on the same day a possible scenario would be active recovery using other exercises that are easier to calculate the accurate work rate.

### Practical implications

Given the complexity of tennis, which involves numerous parameters, players engage in high training volumes from an early age including thousands of repetitions. Over the last decade, tennis has evolved dramatically demanding outstanding court coverage and agility from the players [34]. Optimising training plans to address all key performance aspects is essential for competitive success. Based on the main findings of this study, on-court sessions and weekly training structure should be adapted to the specific training objective. For example, when the focus is on fast and explosive movements, a work-to-rest ratio of at least 2:1 appears necessary to ensure sufficient recovery between drills. This is supported by evidence showing slower phosphocreatine resynthesis following short maximal sprint exercise, highlighting the need for adequate recovery to maintain power output during repeated sprint efforts [35]. To facilitate performance in a subsequent same-day session, 15–20 minutes of active recovery (e.g., cycling) between sessions is recommended, as it reduces blood lactate and supports performance [33]. Consecutive training days should also be organised to accommodate physiological demands. These recommendations are specific to advanced-level adolescent players and may not generalize to professional athletes.

### Limitations

We acknowledge that the current study has a number of limitatons. The study limitations include amongst others the number of participants. In addition, the limited number of participants decrease the effect of players fitness levels and tennis skills that might influence the study results. Furthermore we recognise the challenges in replicating real match conditions, which varies to a great extent and are hard to hard to mimick in a controlled study environment. Also, it should be noted that the 90 s recovery period used in this study likely overestimates the duration of typical between point or changeover games recovery in tennis match play. Therefore, caution should be exercised when generalizing these findings to all forms of tennis recovery. Heart rate was excluded as a variable in this study because it lacks sensitivity to changes in work rate during sports with variable intensity or intermittent character, such as tennis [36,37]. Although heart rate monitoring is a well-established method for assessing internal load, its application in intermittent sports is problematic. Specifically, the commonly used linear relationship between heart rate and $\dot{\text{V}}{\text{O}}_{\text{2}}$, established during incremental laboratory protocols, has been shown to overestimate energy expenditure in such contexts [38,39]. This is because heart rate does not respond as dynamically as oxygen uptake during variable intensity efforts. In tennis heart rate-based intensity zones tend to overestimate the time spent in moderate and high-intensity domains when compared to ventilatory threshold-based methods [1]. We are aware that the exclusion of heart rate data may constrain the comparability of our findings with previous research based solely on heart rate measures.

## Conclusions

Commonly used tennis drills seem more strenuous than previously shown due to the physiological response in between series. Noticeably, oxygen uptake is substantially elevated, and and players exhibit signs of respiratory compensation during recovery periods. This shows that tennis drills can be used to challenge both the aerobic and anaerobic energy systems to a large degree. Furthermore, a work ratio of 3:1 with a 90 s rest in between is not sufficient for blood lactate recovery which should be considered when preparing for these tennis drills practices.
